# Assessment of Land Cover and Land Use Change Dynamics in Kibwezi Watershed, Kenya

**DOI:** 10.1155/2022/3944810

**Published:** 2022-12-12

**Authors:** Reuben C. Ruttoh, John P. O. Obiero, Christian T. Omuto, Lucas Tanui

**Affiliations:** ^1^University of Nairobi, School of Engineering, Department of Environmental and Bio-Sytems Engineering, P.O. Box 30197, Nairobi 00100, Kenya; ^2^Kenya Agricultural and Livestock Research Organization, Kaptagat Road, Loresho, P.O. Box 57811, Nairobi 00200, Kenya

## Abstract

Land use and land cover (LULC) parameters influence the hydrological and ecological processes taking place in a watershed. Understanding the changes in LULC is essential in the planning and development of management strategies for water resources. The purpose of the study was to detect changes in LULC in the Kibwezi watershed in Kenya, using geospatial approaches. Supervised and unsupervised classification techniques using remote sensing (RS) and geographical information system (GIS) were used to process Landsat imagery for 1999, 2009, and 2019 while ERDAS IMAGINE™ 14 and MS Excel software were used to derive change detection, and the Soil and Water Assessment Tool (SWAT) model was used to delineate the watershed using an in-built watershed delineation tool. The watershed was classified into ten major LULC classes, namely cropland (rainfed), cropland (irrigated), cropland (perennial), crop and shrubs/trees, closed shrublands, open shrubland, shrub grasslands, wooded shrublands, riverine woodlands, and built-up land. The results showed that LULC under shrub grassland, urban areas, and crops and shrubs increased drastically by 552.5%, 366.2%, and 357.1% respectively between 1999 and 2019 with an annual increase of 35.55%, 35.38%, and 33.86% per annum. The area under open shrubland and closed shrubland declined by73.7%, and 30.4% annually. These LULC transformations pose a negative impact on the watershed resources. There is therefore a need for proper management of the watershed for sustainable socio-economic development of the Kibwezi area.

## 1. Introduction

The watershed comprises both biotic and abiotic components, including human beings confined within a defined boundary. The watershed provides essential services necessary for the well-being of society [1]. Despite the benefits of watersheds, the quantity and quality of services have been declining over time [2], which are attributed to land use/cover, climate change, and the demand for these services from the rapidly increasing population.

Land use and land cover (LULC) and its resources support for social, cultural, material, and spiritual needs of human beings and have culminated in a significant transformation of the resource [3]. The LULC change on the land surface is noticeable and takes place at a high rate, consequently limiting the potential of natural ecosystems to provide environmental services [4]. The LULC change has been caused by the interaction between demographic, socio-economic, and biophysical changes [5, 6] and is believed to be a major force both locally and globally influencing environmental change [7, 8]. The increasing alteration of the land surface due to anthropogenic activities have greatly affected the effectiveness of global environmental systems.

Rapid LULC change is occurring largely in the developing world [9, 10] with natural vegetation converted to agricultural land [11, 12] which has resulted in a decline in water, soil, and vegetation resources [13]. It affects hydrological processes in the watershed such as evapotranspiration, interception, and infiltration, consequently altering surface and subsurface flows [14, 15]. The LULC through deforestation is believed to be the driver of climate change [7, 8] that could result in loss of biodiversity and consequently have a negative impact on eco-tourism.

The LULC changes originate from a local level, but due to their speed, extension, and intensity, they have various and critical global effects felt on the resources. The ever-increasing change is worrying and can have a significant impact on the local, regional, national, and global environment [16, 17].

The stakeholders, mainly scientists and water planners involved in decision making need the LULC information to determine the change that has occurred in the natural resources [18]. They consider LULC as a process that affects the natural environment and socio-economic situation significantly at local and global conditions. The LULC information helps to understand and extend which human beings have impacted on the natural environment assessment, especially at the watershed level. Conventional methods for capturing and analyzing multidisciplinary spatial data are time-consuming and costly and have been replaced by digital, robust, and effective technologies such as remote sensing (RS) and geographical information systems (GIS). RS and GIS techniques have been used widely in different fields; agriculture [18] and environments [19] (T. Fung and E. Ledrew 1987) and integrated eco-environment assessment. However, many studies have concentrated on LULC due to its extensive impacts on ecology and vegetation [17] with limited studies at a watershed level which have a great impact on the hydrological processes. Soil and Water Assessment Tool (SWAT), a hydrological model, on the other hand, is a GIS interface that analyses raster data from RS to delineate the extent of the watershed based on the outlet details [20]. The SWAT model has been used extensively globally due to its ability to capture the heterogeneity of the watershed by delineating the watershed using the river gauging station outlet.

Makueni County has undergone rapid land cover changes [21] attributed to population increase, urbanization, and agricultural development activities. The changes have resulted from LULC modification over a period of time believed to have increased land degradation and impacted negatively on water resources in the area. The LULC conversion may alter the hydrology of Kibwezi watershed through the intervention of precipitation, surface flow, infiltration, evapotranspiration, and aquifer storage. The study, therefore, aimed at delineating the Kibwezi watershed and comprehensively classifying and detecting the conversion matrix of the LULC using RS, GIS, and SWAT tools. This was achieved through classifying and analyzing LULC using 30 m resolution Lansat imagery for a period of 20 years (1999–2019) and using a 30 m resolution DEM to demarcate and characterize the watershed. It aimed at addressing the following questions: (i) what are the LULC categories present in Kibwezi watershed, (ii) what are the trends in LULC classes within the study period, and (iii) the major conversions in LULC that have taken place in Kibwezi watershed from 1999 to 2019. The results can provide a guide to decision-makers and managers in the layout of structures, monitoring, and management of the watershed.

## 2. Materials and Methods

### 2.1. Study Area

#### 2.1.1. Location

**Figure 1:**
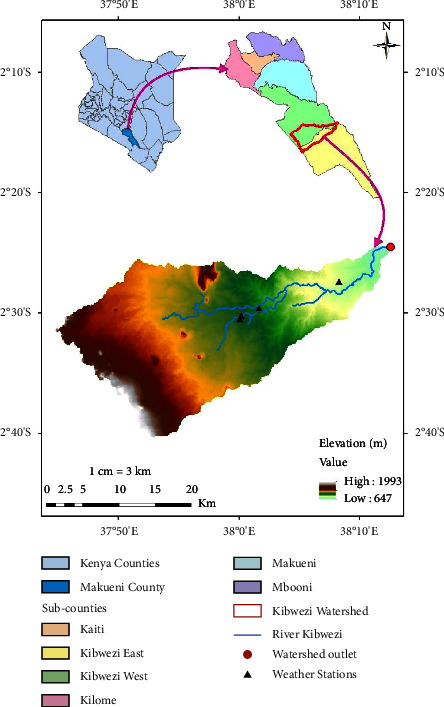
Kibwezi watershed location.

Kibwezi watershed is located in Semi-Arid Eastern Kenya, Makueni County, in the lower part of the River Athi basin as presented in Figure 1. The watershed spans an area of approximately 700 km^2^ in Kibwezi west and Kibwezi east subcounties and is described by the co-ordinates 37.73°E to 38.15°E and 2.32°N to 2.58°N at an altitude between 647 m and 1993 m above sea level.

#### 2.1.2. Climate

Kibwezi watershed typically semiarid climate characterized by varied and erratic rainfall. It receives a bi-modal type of rainfall; March–May for long rains, and November–December for short rains with an annual average of 600 mm, while the minimum and maximum temperatures are 17°C and 27°C, respectively [22]. River Kibwezi is the main distributary in the Kibwezi watershed and is approximately 25 km long. The watershed is also characterized by flat topography with a deposit of alluvial soil along the river valleys and sandy soils in the plain. Shallow to moderately deep soils are common and the landscape is bordered by the Yatta plateau on the eastern side.

The prevalent vegetation of the Kibwezi watershed is savanna and shrub-woody vegetation with various species. It varies according to rainfall amount, soil type, and based on the species of grass or shrubs.

### 2.2. Data Acquisition and Preparation

#### 2.2.1. Land Use/Land Cover Maps

**Figure 2:**
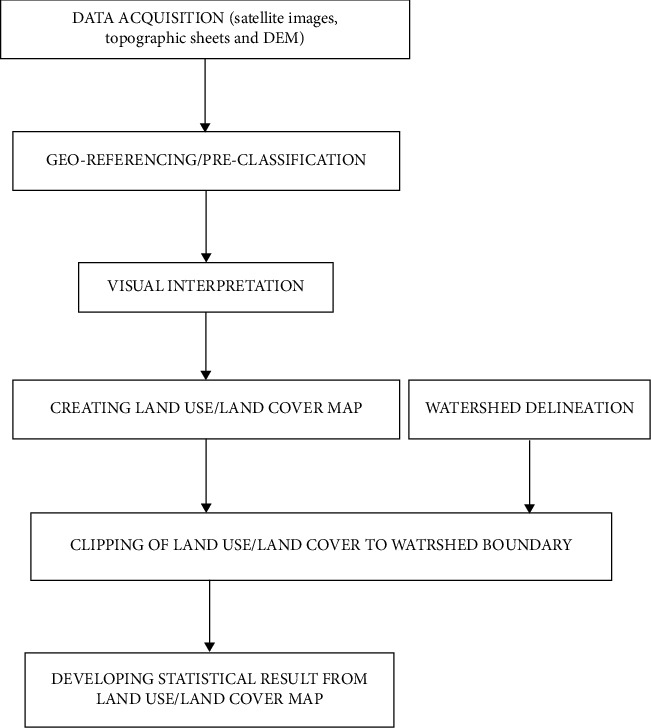
Flowchart of methodology.

*(1) Preparation of Field Base Maps*. Landsat images covering the Kibwezi watershed were obtained from the USGS Global Visualization Viewer (https://glovis.usgs.gov). The Landsat images were obtained in raster format at 30 m × 30 m resolution and were extracted for a ten-year interval from 1999 to 2019 for the study area. The Landsat TM5 was available for the period 1999 and 2009 while Landsat 8 satellite imagery was available for 2019. The images obtained were clear and free from cloud cover. The individual spectral bands that were downloaded from the website were stacked together to form multispectral imagery scenes for the three periods, and thereafter subsets of the watershed (area of interest) were extracted from each of the multispectral Landsat scenes. The digital image processing software ERDAS IMAGINE™ 14 and ArcGIS 10.2 were used for the processing, analysis, and integration of spatial data to arrive at the objectives of the study. The LULC processes development, included data acquisition preclassification to identify possible cover polygons, ground truthing to validate LULC classes, and development of final LULC maps (Figure 2). The imageries were terrain-corrected (ortho-rectified) and hence were directly used for classification and GIS analysis. The characteristics of satellite imagery are presented in Table 1.

**Figure 3:**
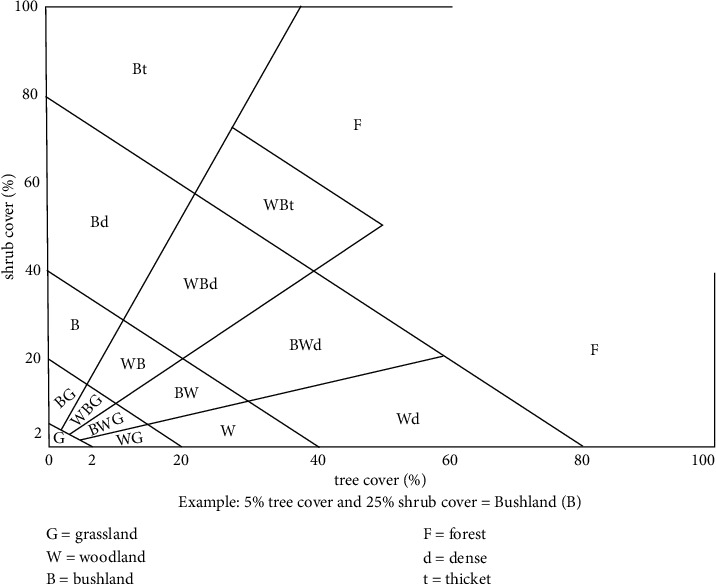
Key to physiognomic classes.

The ERDAS IMAGINE was used to perform ISODATA unsupervised classification on the 2019 imagery with 12 clusters/classes which were then classed using a minimum-distance classifier. Once the initial classification was completed, the 12 spectral classes were assigned to the 10 information classes in Table 2 based on the Kenya Soil Survey key to physiognomic classes presented in Figure 3. As with almost any automated classification technique, the initial raw land cover maps were characterized by speckling produced by the misclassification of a single pixel or small groups of pixels and hence the need for cartographic generalization. To eliminate much of the visual noise caused by these misclassified pixels, the raw maps were converted from ERDAS raster format to ArcGIS shape file and generalized using a series of both automated and manual procedures. The eliminate tool in the ArcGIS ArcToolbox was used to remove the small sliver polygons by merging them with adjacent polygons. The remaining coded polygons were the preliminary land cover polygons.

**Figure 4:**
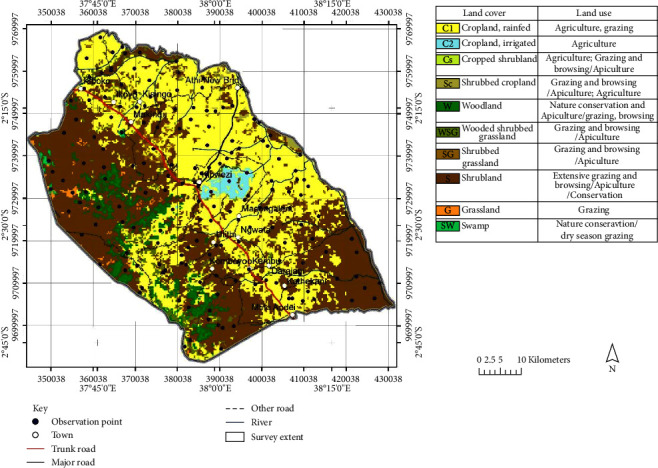
Preclassified land cover map.

The same procedure was also adopted to generate the 1999 and 2009 land use/cover maps. The existing digital thematic data on roads, rivers, administrative boundaries, and settlements from the KSS GIS database were projected to WGS 84 UTM zone-37 south coordinate system, which was adopted for the study. These data together with preliminary land cover polygons and randomized observation points were overlaid on the Landsat 8 imagery of 2019 to produce a field base map as shown in Figure 4. A preliminary legend was also constructed on the basis of the different land cover classes. This map together with GPS and digital Google Earth images assisted in the location of sites and was vital for the ground truthing and postclassification process.

*(2) Field Data Collection Methods*. The unsupervised image classification method was carried out prior to the field visit to determine the strata for ground truthing. The aim of collecting data was to validate land use and land cover interpretation from the satellite images of 2019 and for a qualitative description of the characteristics of each land use/land cover. A random sampling procedure was applied to ensure representative samples in all preclassified LULC polygons within the watershed. The number of samples per LULC class was determined based on the size of the unit and they were equidistant from one another.

The determination of position and general orientation was carried out using a Trimble SB hand held GPS receiver. The final position data were recorded once the GPS's position dilution of precision (PDOP) values were less than three considering the land cover units were derived from Landsat imagery with a spatial resolution of 30 meters. Each time a randomized observation point was approached, a brief description of the land use and land cover observed (percent aerial cover of trees, shrubs, herbs, grasses, bare soil, and others) were recorded on the field observation forms and geographical coordinates were saved into the GPS-databank and photographs. The dominant physiognomic classes (structure) of the vegetation were also recorded. Information on dominant plant species was also recorded. October and November mark the rainy season in lower eastern Kenya and therefore images were chosen during this season in order to best distinguish the spectral signatures of the land cover types since the vegetative growth is at its peak. Also, the near-anniversary dates were chosen for consistency between the two time points.

*(3) Postclassification*. The image interpretation boundaries which needed adjustment were adjusted accordingly to the base map with reference to field observations. The gathered information on sampling points, tracking, and waypoints were used to finalize the land cover codes used to describe each mapping unit depending on the land use/land cover class. The land cover maps for 1999 and 2009 were converted from vector format to raster format using the ArcGIS software. The land cover maps were then recorded into 9, 10, and 10 broad land cover classes respectively (i.e., built-up land, wooded shrublands, etc.), for GIS overlay analysis for change statistics and map production.

The LULC maps for the Kibwezi watershed were prepared by clipping the mask area of the Kibwezi watershed using ArcGIS for the period 1999, 2009, and 2019.

### 2.3. Accuracy Assessment

Accuracy assessment in LULC is crucial for the classified classes before embarking on change detection (Owojori, A and Xie, H. 2005). It assists to assess the quality of data collected in the field compared to the classified satellite images to identify any error and its source. Ground thruthing of LULC classes were compared to results from satellite images Landsat TM5 and Landsat 8. In this study, 547 pixel points were produced during LULC classification guided by a minimum sample size of 50 per LULC class [23]. Accuracy was assessed based on visual interpretation and the ground-truth data and the overall results were obtained by dividing the sum of the correctly classified LULC pixels classes in each sampled unit and a total number of reference pixels which are then captured in the error matrix. The Kappa test, a nonparametric statistic based on a discrete multivariate technique was used to determine the magnitude of accuracy between referenced and imagery data. Manomani and Suganya [24] have categorized Kappa statistics into six categories; less than 0 indicating no agreement, 0–0.2 indicates slight agreement, 0.0–0.41 is poor, 0.41–0.60 showing moderate, 0.60–0.80 as significant, and 0.81–1.0 indicating perfect agreement of referenced versus image data.

### 2.4. LULC Change Detection

The LULC change maps were developed for the period 1999–2009 and 2009–2019 to help understand the changes in the Kibwezi watershed. The amount of LULC class, the percentage of change, and the annual rate of changes for the periods were calculated using the following equations:

(1) $$\begin{array}{c} Change=A_{j}-A_{i}\text{,}\\ \%\;Change=\left(\frac{A_{j}-A_{i}}{A_{i}}\right)*100\text{,}\\ Annual\;rate\;\%=\left(\frac{A_{j}-A_{i}}{A_{i}*n}\right)*100\text{.} \end{array}$$

Where *A*_*i*_ is the initial LULC class area (ha), *A*_*j*_ is the final LULC class area (ha), and *n* is the number of years between the initial and final time period.

### 2.5. Kibwezi Watershed DEM

**Figure 5:**
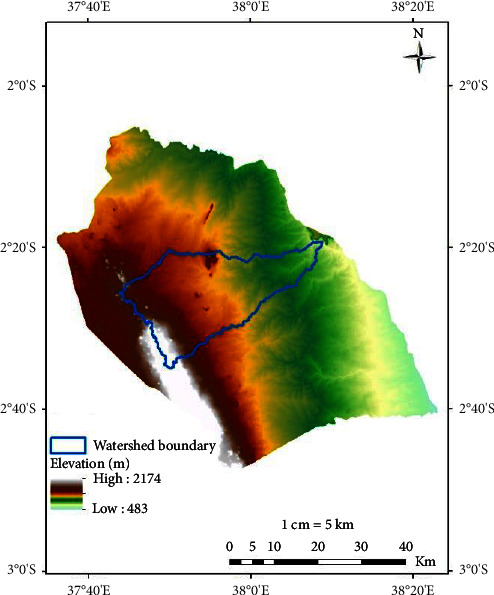
DEM.

**Figure 6:**
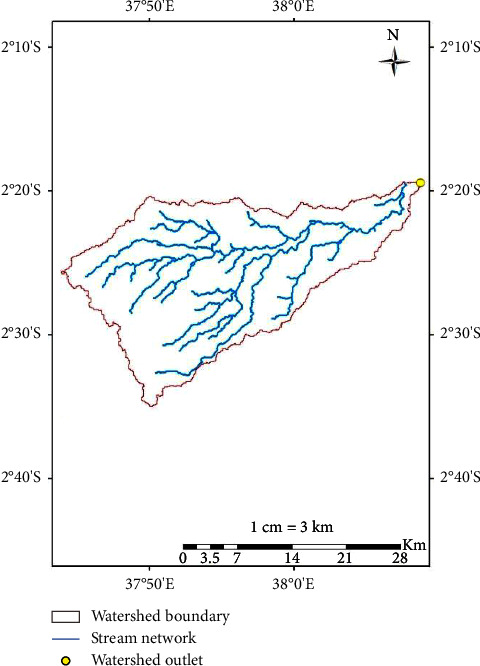
Watershed.

A Shuttle Radar Topographic Mission, a digital elevation model (SRTM DEM) with a resolution of 30 m and covering the extent of the watershed was obtained from Kenya Soil Survey (KSS). The DEM represents the digital topography image of the mask area of the Kibwezi watershed. The DEM was clipped and projected to WGS 84 UTM zone-37 south using ArcGIS to cover the Kibwezi watershed boundary as shown in Figure 5. Using the DEM as input and the River Kibwezi discharge point to River Athi as an outlet, the SWAT model [20] was used to delineate the watershed presented in Figure 6.

## 3. Results

### 3.1. Accuracy Assessment

The overall LULC classification accuracy for the Kibwezi watershed ranged between 64.3% and 100.0% for user accuracy (CA) while producer accuracy (PA) ranged between 59.1% and 100.0% for individual classes (Table 3). The overall assessment accuracy was 87.4% while the Kappa coefficient was 0.85. The summary for individual accuracies is shown in the table. From the results, the Kappa values for the LULC classification are over 80 and thus qualified as a very significant predictor [24]. The LULC accuracy provides a basis for subsequent analysis of the changes in the watershed.

### 3.2. Land Use/Land Cover Map from 1999 to 2019

**Figure 7:**
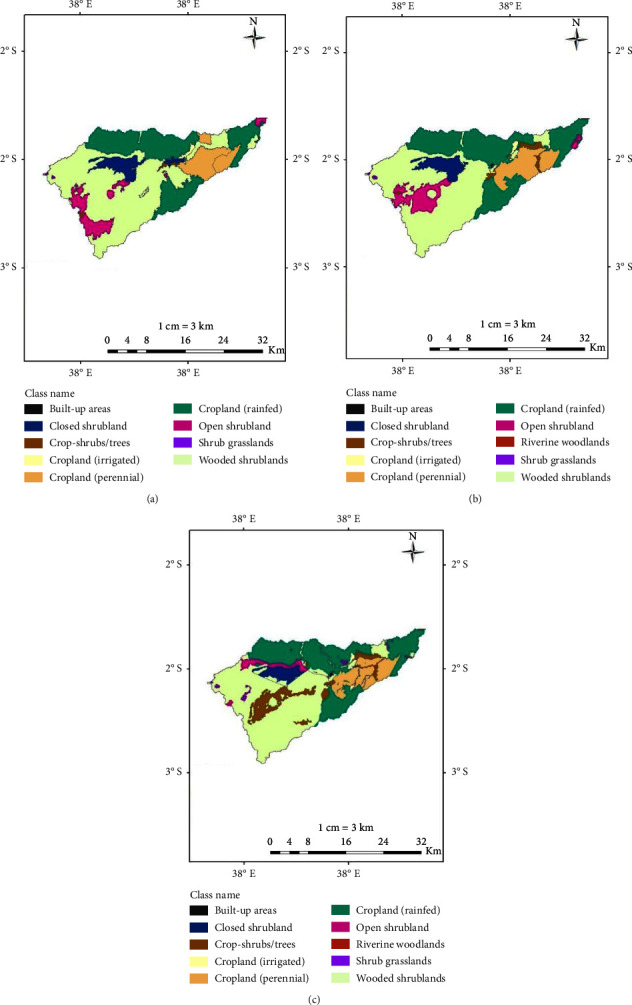
(a) LULC map in 1999. (b) LULC in map 2009. (c) LULC map in 2019.

The results of the multitemporal satellite image classification showed that the total area of the Kibwezi watershed was 61,490 ha (614.9 km^2^). The watershed was classified into ten LULC classes; (i) built-up areas, (ii) cropland (rainfed), (iii) cropland (irrigated), (iv) cropland (perennial), (v) cropshrubs/trees, (vi) closed shrubland, (vii) open shrubland, (viii) shrub grassland, (ix) wooded shrubland, and (x) riverine woodlands. The LULC were identified and analyzed for the periods 1999, 2019, and 2019. The individual LULC classes for 1999, 2009, and 2019 periods are shown in Figures 7(a)–7(c) and summarized in Table 4.

The percentage area of each LULC class in 1999, 2009, and 2019 indicated that wooded shrublands and cropland (rainfed) occupied the largest share of the watershed, representing on average 45% and 30% respectively across the period. The classification also revealed that the area under built-up land, cropland (irrigated), shrub grasslands, and riverine woodlands each occupied less than 1% of the total watershed across the period.

### 3.3. Land Cover Change Detection 1999–2019

Tables 5 and 6 show the LULC transition matrix from one class category to another by hectares between 1999/2009 and 2009/2019. In Table 5, the results show that between 1999 and 2009, approximately 2,239 ha were converted to cropland (rainfed) comprising 958 ha of cropland (irrigated, perennial, and crops/shrubs), 378 ha of closed shrubland, 256 ha of open shrublands, and 994 ha of wooded shrublands. In addition, 140 ha of cropland (rainfed), 14 ha of crops and shrubs/trees, a total of 1,001 ha of shrubland areas (closed, open, and wooded shrublands) were converted to cropland (perennial). In the same period, 2,970 ha of wooded and closed shrubland were converted to open shrubland, while a total of 4,495 ha of closed and open shrubland were converted to wooded shrubland.

Table 6 shows that between 2009 and 2019, approximately 58 ha of cropland (rainfed), 25 ha of cropland (perennial), and 32 ha of wooded shrubland were converted to built-up land or urban, while a total of 184 ha of cropland (irrigated and perennial) and crops and shrubs/trees were converted to cropland (rainfed). Furthermore, the area under rainfed crops (248 ha) and crops-shrubs/trees (34 ha), 1 ha under wooded shrublands, and 7 ha under riverine woodland changed to cropland (perennial) land. In addition, a total of 489 ha of cropland (rainfed, irrigated, and perennial) and 3437 ha of both open and wooded shrubland were transformed to crops-shrubs/trees land during this period. The cropland (rainfed, perennial, and crops-shrubs/trees) lost 770 ha, while open shrubland was reduced by 1,675 ha to wooded shrubland in 2009–2019 period.

Table 7 summaries the dynamic changes of different land use/land cover from 1999 to 2019. The results show a continuous increase of area under built-up land, shrub, grassland, and crops-shrubs/trees land, while the area under open and closed shrubs was on a decline. The biggest increment between 1999 and 2019 was the area under crops-shrubs/trees (3,606.5 ha), followed by cropland (rainfed) (1,206.5 ha), shrub grasslands (359.7 ha), riverine woodlands (349.2 ha), and built-up land (116.2 ha). Open shrublands area decreased by 4423 ha, followed by closed shrublands (963.3 ha) and wooded shrublands (177.2 ha), while cropland (perennial and irrigated) declined by 44.8 ha and 29.9 ha, respectively, as shown in the table. On the other hand, the area under cropland (perennial) and cropland (rainfed) increased between 1999 and 2009 but decreased between 2009 and 2019, while the area under wooded shrublands and cropland (irrigated) was reverse.

Moreover, from the results, the rate of expansion in shrub grasslands was the highest, followed by built-up land, and crops-shrubs/trees land, while the lowest expansion was in open and closed shrublands between the overall periods from 1999 to 2019.

## 4. Discussion

Global environment has undergone tremendous change that significantly influences hydrological processes in the watershed resulting to hydrologic nonstationarity. The LULC is believed to contribute significantly to change in global environment [25]. Remote sensing and GIS tools and integration with SWAT model have proved to be efficient, accurate, and cost-effective and give detailed information to analyze, detect, and monitor LULC changes at the watershed level. The Kibwezi watershed has witnessed great LULC as shown in the transition matrix (Table 7). In the watershed, ten LULC classification classes, built-up areas, cropland (rainfed), cropland (irrigated), cropland (perennial), crops-shrubs/trees, closed shrubland, open shrubland, shrub grassland, wooded shrubland, and riverine woodlands, were identified and analyzed for the periods 1999, 2019, and 2019. The LULC change between 1999 and 2019 indicates that riverine woodlands areas increased by 349.2 ha within the period. The shrub grassland, urban areas, crops-shrubs, and rainfed cropland land covers had a positive percentage indicating an increase of 552.5%, 366.2%, 357.1%, and 7.0% respectively. Open shrubland, closed shrubland, cropland (irrigated), cropland (perennial), and wooded shrublands had a negative percentage cover of 73.7%, 30.4%, 10.4%, 0.8%, and 0.6%, respectively.

The results show that there is a great conversion of natural vegetation into cropland and built-up areas in the last 20 years in the watershed. A reduction in shrubs is associated with the expansion in urbanization within the study area. The Kibwezi town and other trading centres are expanded due to commercial and business activities. The urbanization is also attributed to increase in the population due to the growth rate in the area and sisal commercial farm within the watershed which provide employment. According to [26], population growth rate of Kibwezi stands at 1.4 per cent per year and a population density of 121 persons per square kilometer. This increase in population has led to a high demand for expansion of land through deforestation for the production of cereals, legumes, and horticultural crops for the population and also for commercial purposes. This growth has led to the clearing of shrubs for built-up area and also for agricultural activities. The findings are similar to [12, 27], where population growth and GDP plays a big role in LULC conversion.

Table 7 shows that open and closed shrublands have decreased by 5386 Ha while cropland increased by 3606.5 ha between 1999 and 2019 indicating a huge decline in the vegetation cover. This decline is attributed to encroachment of the rising population to the watershed despite the land use policy in this place. The poor enforcement of land use policy may have resulted in decreased vegetation, consequently increased surface runoff, and reduction in recharge of the watershed that has led to the drying of the distributaries in the watershed. In addition, riverine woodlands have increased significantly during the study period, which may have been as a result of deposition of sediments from the upstream. The Kibwezi area has been experiencing occasional drought where the rainfalls have been erratic and temperatures increasing [28]. As a meaning of livelihood under this climate change, the population has been forced to clear the existing shrubs for timber, firewood, and charcoal for domestic and commercial use leading to land cover conversion.

## 5. Conclusions

The use of RS and GIS has demonstrated the effectiveness of spatial digital techniques in detecting, assessing, and monitoring the LULC in the Kibwezi watershed. The Kibwezi watershed was classified into built-up areas, croplands, crops-shrubs/trees, shrublands, and riverine woodlands of LULC classes. The different class categories underwent varied changes with some maintaining a constant increase or decreases in the area during the 1999/2009 and 2009/2019 period. Overall, the results showed that LULC under shrub grasslands, urban areas, crops-shrubs, and rainfed croplands increased by 552.5%, 366.2%, 357.1%, and 7.0%, respectively, between 1999 and 2019 while LULC under open shrubland, closed shrubland, cropland (irrigated), cropland (perennial), and wooded shrublands decreased by 73.7%, 30.4%, 10.4%, 0.8%, and 0.6%, respectively, during the same period. The shrub grassland, urban areas, and crops and shrubs had an annual increase of 35.55%, 35.38%, and 33.86%, respectively, while open shrubland and closed shrubland declined by 73.7%, and 30.4% per annum. The increasing rate of urban areas and cropland at the expense of vegetation will influence land degradation, runoff, and ground water recharge and results in decreased water and food productivity. These LULC transformations can assist the watershed managers and policy makers in developing management strategies for the watershed for sustainable socio-economic development of the Kibwezi area. Future studies in the watershed should analyze the impact of climate change and management practices on the watershed hydrology to guide in improving the water and soil productivities in the Kibwezi area.

## Figures and Tables

**Table 1:** Characteristics of the remotely sensed data.

Satellite	Sensor	Spatial/spectral resolution	Acquisition date	Path/row
Landsat 5	Thematic mapper (TM)	30 m (VIS, NIR bands)	25/10/99	167/062
Landsat 5	Thematic mapper (TM)	30 m (VIS, NIR bands)	13/11/09	167/062
Landsat 8	Operational land imager (OLI)	30 m (VIS, NIR, SWIR bands)	08/10/19	167/062

**Table 2:** LULC class and classification definitions.

Class name	Description
Built-up land	Areas characterized by close settlements for residential, industrial, commercial, and public service
Cropland (rainfed)	Areas characterized by vegetation planted under rainfed conditions for the production of food, feed, or fibre. Crops include pigeon peas, green grams, cowpea, beans, maize, mango, and miraa (under irrigation). Field edges and uncultivated areas are dominated by *Acacia Vachellia elatior* sp.
Cropland (irrigated)	Areas characterized by vegetation under irrigation for production of food, feed, or fibre. Main crops are tomatoes, maize, assorted vegetables, and mangoes
Cropland (perennial)	Areas under rainfed condition for sisal production
Crops-shrubs/trees	These are areas characterized by woody vegetation (>50%) *Acacia Vachellia elatior* sp. as well as growing of crops under rainfed condition (<50%) pigeon peas, maize, and beans
Closed shrublands	Areas characterized by woody vegetation generally less than 6 m tall (>80%). Trees cover 5% and shrubs cover >80% and bare 60%. Dominant woody species are as in unit shrub grasslands (SG)
Open shrublands	Areas characterized by woody vegetation are generally less than 6 m tall (20–49%). Over 80% of the area is bare and very degraded due to lack of basal cover. The shrub cover ranges from 20 to 40%. Dominant woody species are as in unit SG
Shrub grasslands	Areas dominated by grasses and herbs (>60%) and some shrubs (<30%). The dominant grass species are *Digitaria macroblephara, Chloris roxburghiana, and Sporobolus pellucidus*, while the woody species are *Commiphora campestris and, Acacia Vachellia elatior*
Wooded shrublands	Areas dominated by shrubs with a tree cover of <20%. A very sparse herb layer is present only in a few places. Among the grasses are *Enteropogon macrostachyus* and *Penicum coloratum*, while the herbs are *Triumfetta flavescens* and *Ipomea lapidosa.* The dominant trees are *Commiphora baluensis* and *Melia volkensii* while shrubs are *Cordia ovata* and *Croton dichogamus* characterized by lava flows and rock outcrops
Riverine woodlands	They are found along river alluvial plains with drainage channels (river valleys) covered by trees and shrubs. *Acacia* v*achellia elatior* is the dominant tree and forms a cover of 5–10%. *Balanites aegytiaca and Salvadora persica* (tooth brush). Dominant grasses are *Pennisetum mezianum* and *Sprobolus helvolus*. The areas are heavily grazed due to the proximity of many water holes in the riverbed

**Table 3:** Accuracy assessment MATRIX for LULC classes.

	Classification result												
	LULC classes	BA	*C*1	*C*2	*C*3	CS	cS	oS	SG	WS	RW	Reference points	PA (%)
Ground truth	BA	17	0	0	0	0	0	0	0	0	0	17	100.0
	*C*1	0	65	4	2	1	0	0	0	0	0	72	91.6
	*C*2	0	0	9	5	0	0	0	0	0	0	14	69.2
	*C*3	0	0	0	15	0	0	0	0	0	0	15	68.2
	CS	0	1	0	0	26	0	0	0	0	0	27	59.1
	cS	0	0	0	0	6	67	0	0	0	0	73	93.1
	oS	0	0	0	0	8	0	50	0	0	0	58	90.9
	SG	0	3	0	0	3	5	5	85	12	0	113	92.4
	WS	0	2	0	0	0	0	0	7	136	2	147	90.1
	RW	0	0	0	0	0	0	0	0	3	8	11	80.0
	Classified points	17	71	13	22	44	72	55	92	151	10	547	
	CA (%)	100.0	90.3	64.3	100.0	96.3	91.8	86.2	75.2	92.5	72.7		
	Overall accuracy							87.4					
	Kappa coefficient							0.85					

LULC category codes: BA = built-up area; *C*1 = cropland (rainfed); *C*2 = cropland (irrigated); *C*3 = cropland (perennial); cS = cropshrubs/trees; cS1 = closed shrubland; oS = open shrubland; SG = shrub grassland; WS = wooded shrubland; RW = riverine woodlands.

**Table 4:** LULC class transitions 1999–2019.

Class name	1999		2009		2019	
	Area (ha)	Area (%)	Area (ha)	Area (%)	Area (ha)	Area (%)
BA	31.7	0.1	33.7	0.1	147.8	0.2
C1	17258.6	28.1	19498.0	31.7	18465.0	30.0
C2	285.6	0.5	221.2	0.4	255.8	0.4
C3	5719.3	9.3	5811.4	9.5	5674.5	9.2
cS	1010.0	1.6	1013.6	1.7	4616.5	7.5
cS1	3164.2	5.2	2957.6	4.8	2200.9	3.9
oS	5997.9	9.8	4282.6	7.0	1574.9	2.6
SG	65.1	0.1	93.6	0.2	424.8	0.7
WS	27957.8	45.5	27516.1	44.8	27780.6	45.2
RW		0.0	62.4	0.1	349.2	0.6
Total	61490	100	61490	100	61490	100

LULC category codes: BA = built-up area; *C*1 = cropland (rainfed); *C*2 = cropland (irrigated); *C*3 = cropland (perennial); cS = cropshrubs/trees; cS1 = closed shrubland; oS = open shrubland; SG = shrub grassland; WS = wooded shrubland; RW = riverine woodlands.

**Table 5:** LULC change matrix for the period 1999–2009 by hectares.

	Class name	1999										
		BA	*C*1	*C*2	*C*3	cS	cS1	oS	SG	WS	RW	Total
2009	BA	32										32
	*C*1		16911	78	140	10		27		94		17259
	*C*2		127	135		18				6		286
	*C*3		759		4658	217				24	62	5719
	cS	0	72		14	1		33		889		1010
	cS1		378		5		2611	1		169		3164
	oS		256		74	55	2	1285		4326		5998
	SG								55	10		65
	WS	2	994	9	922	713	345	2937	38	21998		27958
	Total	34	19498	221	5811	1014	2958	4283	94	27516	62	61490

LULC category codes: BA = built-up area; *C*1 = cropland (rainfed); *C*2 = cropland (irrigated); *C*3 = cropland (perennial); cS = crops-shrubs/trees; cS1 = closed shrubland; oS = open shrubland; SG = shrub grassland; WS = wooded shrubland; RW = riverine woodlands.

**Table 6:** LULC change matrix for the period 2009–2019 by hectares.

	Class name	2009										
		BA	C1	C2	C3	cS	cS1	oS	SG	WS	RW	Total
2019	BA	33	0									34
	*C*1	58	17933	92	248	394		45	148	543	37	19499
	*C*2	0	70	135		3				0	12	221
	*C*3	25	92	23	5384	92				22	174	5811
	cS	0	22	1	34	690			1	205	61	1014
	cS1					0	2135	56		767		2958
	oS		180			2293		1	134	1675		4283
	SG								74	20		94
	WS	32	169	5	1	1144	66	1473	68	24548	10	27516
	RW				7	0				0	55	62
	Total	148	18465	256	5675	4616	2201	1575	425	27781	349	61490

LULC category codes: BA = built-up area; *C*1 = cropland (rainfed); *C*2 = cropland (irrigated); *C*3 = cropland (perennial); cS = crops-shrubs/trees; cS1 = closed shrubland; oS = open shrubland; SG = shrub grassland; WS = wooded shrubland; RW = riverine woodlands.

**Table 7:** LULC changes from 1999 to 2019.

Class name	1999		2009		2019		Change (1999–2019)		Annual rate of change (%)	
	Area (ha)	Area (%)	Area (ha)	Area (%)	Area (ha)	Area (%)	Area (ha)	Area (%)	1999–2009	2009–2019
Built-up land	31.7	0.1	33.7	0.1	147.8	0.2	116.1	366.2	0.63	33.86
Cropland (rainfed)	17258.6	28.1	19498.0	31.7	18465.0	30.0	1206.4	7.0	1.30	−0.53
Cropland (irrigated)	285.6	0.5	221.2	0.4	255.8	0.4	−29.8	−10.4	−2.25	1.56
Cropland (perennial)	5719.3	9.3	5811.4	9.5	5674.5	9.2	−44.8	−0.8	0.16	−0.24
Crops-shrubs/trees	1010.0	1.6	1013.6	1.6	4616.5	7.5	3606.5	357.1	0.04	35.55
Closed shrublands	3164.2	5.1	2957.6	4.8	2200.9	3.6	−963.3	−30.4	−0.65	−2.56
Open shrublands	5997.9	9.8	4282.6	7.0	1574.9	2.6	−4423.0	−73.7	−2.86	−6.32
Shrub grasslands	65.1	0.1	93.6	0.2	424.8	0.7	359.7	552.5	4.38	35.38
Wooded shrublands	27957.8	45.5	27516.1	44.7	27780.6	45.2	−177.2	−0.6	−0.16	0.10
Riverine woodlands	—		62.4	0.1	349.2	0.6	—	—	—	45.96
Total	61490.2	100.0	61490.2	100.0	61490.2	100.0				

## Data Availability

The data that support the research findings of the land use/land cover (LULC) in the Kibwezi watershed are included within the article. However, a supplementary file is attached, which indicates the land cover category codes and their land use description for the same. It also provides LULC for the period 1999, 2009, and 2019 with their respective areas/size in hectares for the study area.

## Supplementary Materials

Supplementary Materials The data that supports the research findings of the land use/land cover (LULC) in the Kibwezi watershed are included within the article. However supplementary file is attached which indicates the land cover category codes and their land use description for the same. It also provides LULC for the period 1999, 2009 and 2019 with their respective areas/size in hectares for the study area.
